# “You gotta lift to get ripped”: injection lift myotomy

**DOI:** 10.1055/a-2317-5012

**Published:** 2024-06-05

**Authors:** Bianca L. Di Cocco, Donevan R. Westerveld, David Carr-Locke, Kartik Sampath, Reem Z. Sharaiha, SriHari Mahadev

**Affiliations:** 1 Division of Internal Medicine, New York Presbyterian Hospital/Weill Cornell Medical Center, New York, United States; 2 Division of Gastroenterology and Hepatology, New York Presbyterian Hospital/Weill Cornell Medical Center, New York, United States

Over the past several years, peroral endoscopic myotomy (POEM) has emerged as a first-line, minimally invasive therapy for achalasia 1 . In a traditional POEM procedure, injection is used to facilitate entry and dissection of a tunnel in the submucosal space 2 . Subsequently, a selective myotomy of the circular and variably longitudinal muscle fibers is performed with limited visualization of structures deep into the muscularis layer. Risks of the myotomy portion of POEM include injury to extraluminal structures and blood vessels, which can lead to pneumothorax, capnoperitoneum, and hemorrhage 3 . In this video, we present a novel technique that employs injection through the muscularis propria layer during the myotomy phase to facilitate a safer and more controlled myotomy ( Video 1 ).

Injection lift myotomy: a new technique for peroral endoscopic myotomy.Video 1

A 26-year-old man presented with a 20-lb weight loss and intermittent episodes of dysphagia to solid foods. Manometry studies were consistent with type II achalasia and he was referred for POEM. The procedure was initiated in the standard fashion using an injection-capable knife ( Fig. 1 ). Submucosal tunneling was extended to 3 cm distal to the gastroesophageal junction ( Fig. 2 ). Novel injection-lift myotomy was then initiated by injecting directly into the muscular propria layer, expanding the intermuscular and subadventitial spaces with the mixture of saline and methylene blue. This allowed for better visualization of the muscle fibers, vessels, and extraluminal structures ( Fig. 3 ). Selective myotomy of the circular muscle bundle was then performed, using intermittent fluid injection to maintain expansion of the subadventitial and intermuscular spaces ( Fig. 4 ). Following the circular myotomy, the distal portion of the longitudinal muscle was divided, resulting in a full-thickness myotomy ( Fig. 5 ). The myotomy was completed without adverse events, with excellent visualization being maintained throughout.

**Fig. 1:**
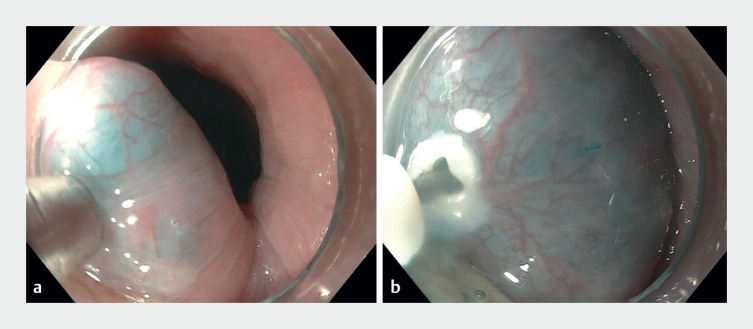
Endoscopic images. **a** A mixture of saline and methylene blue was injected into the submucosa as in a standard peroral endoscopic myotomy procedure. **b** An incision was then created to enter the submucosal space.

**Fig. 2:**
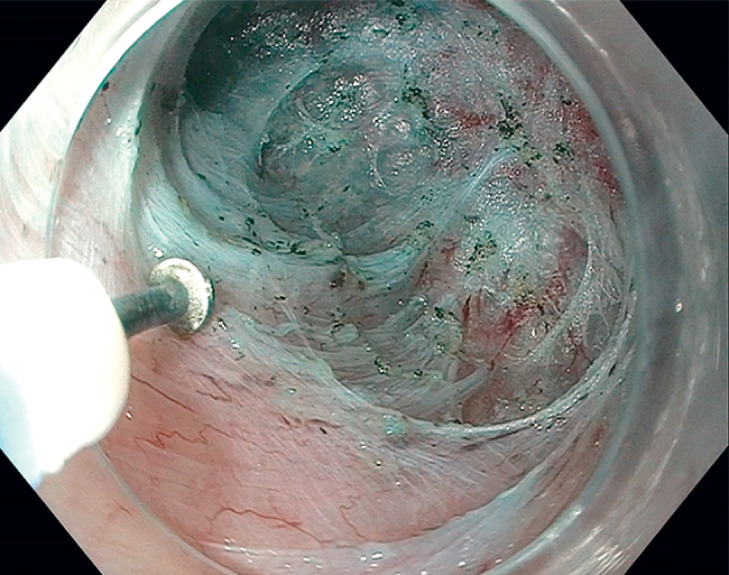
A submucosal tunnel was created, reaching 3 cm distal to the gastroesophageal junction.

**Fig. 3:**
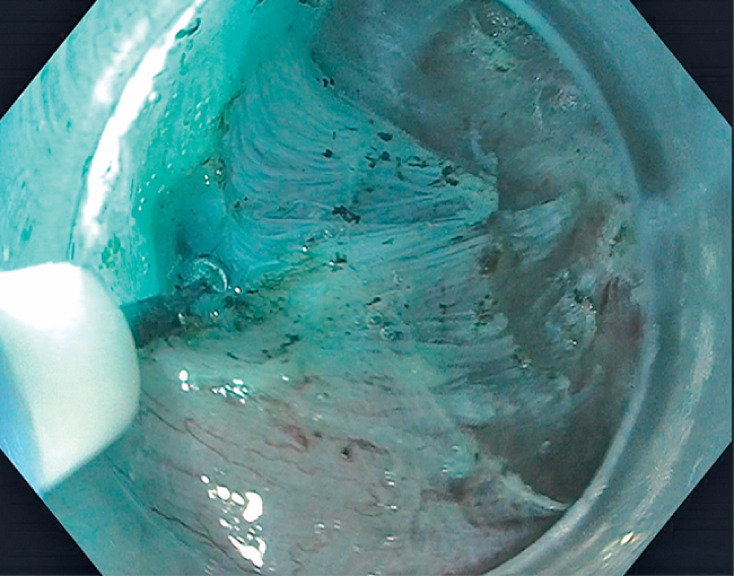
In the injection lift myotomy technique, a mixture of saline and methylene blue is injected into the muscular propria layer, allowing for better visualization of muscle fibers and vessels.

**Fig. 4:**
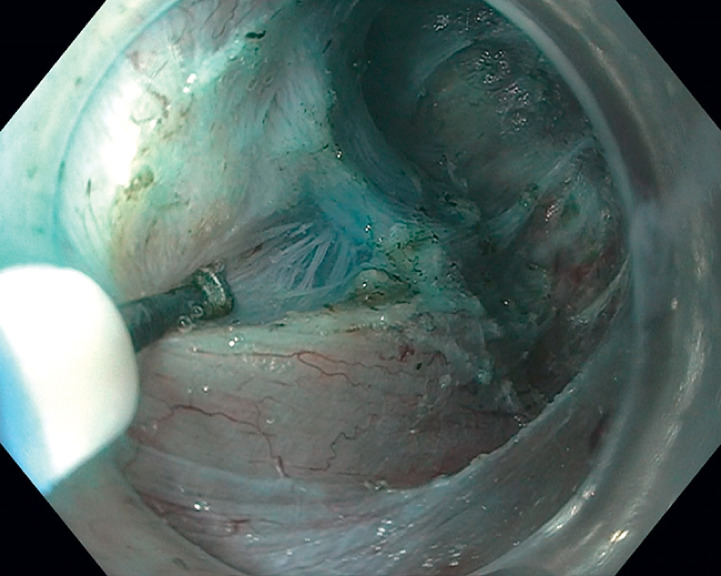
Selective myotomy of the circular muscle bundle was then performed.

**Fig. 5:**
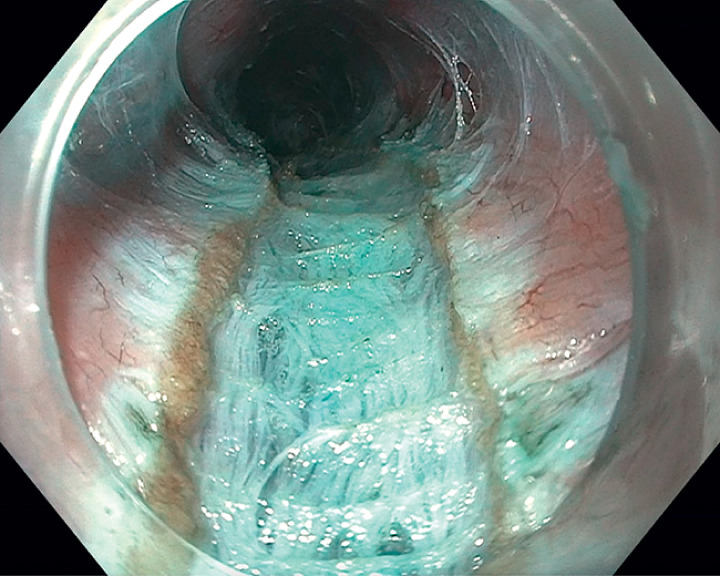
The results after selective circular myotomy.

This case shows how injection-lift myotomy is a safe technique that could potentially reduce the risk of esophageal perforation, bleeding, and damage to adjacent organs during POEM.

Endoscopy_UCTN_Code_TTT_1AO_2AF

## Notes

Endoscopy E-Videos https://eref.thieme.de/e-videos
*E-Videos* is an open access online section of the journal *Endoscopy* , reporting on interesting cases and new techniques in gastroenterological endoscopy. All papers include a high-quality video and are published with a Creative Commons CC-BY license. Endoscopy E-Videos qualify for HINARI discounts and waivers and eligibility is automatically checked during the submission process. We grant 100% waivers to articles whose corresponding authors are based in Group A countries and 50% waivers to those who are based in Group B countries as classified by Research4Life (see: https://www.research4life.org/access/eligibility/ ). This section has its own submission website at https://mc.manuscriptcentral.com/e-videos .
